# Midterm outcomes of midfoot and hindfoot arthrodesis with strut allograft for Müller–Weiss disease

**DOI:** 10.1186/s12891-022-05629-7

**Published:** 2022-07-27

**Authors:** Tung-Ying Lee, Chang-Chin Wu, Kai-Chiang Yang, Kuang-Ting Yeh, Ing-Ho Chen, Chen-Chie Wang

**Affiliations:** 1 Department of Orthopedic Surgery, Taipei Tzu Chi Hospital, Buddhist Tzu Chi Medical Foundation, New Taipei City, 23142 Taiwan; 2 Department of Orthopedics, En Chu Kong Hospital, New Taipei City, Taiwan; 3 Departments of Biomedical Engineering, Yuanpei University of Medical Technology, Hsinchu City, Taiwan; 4 School of Dental Technology, College of Oral Medicine, Taipei Medical University, Taipei, Taiwan; 5 Department of Orthopedic Surgery, Hualien Tzu Chi Hospital, Buddhist Tzu Chi Medical Foundation, Hualien, Taiwan; 6 Department of Orthopedics, School of Medicine, Tzu Chi University, Hualien, 97004 Taiwan

**Keywords:** Müller, Weiss disease, Strut allograft, Arthrodesis, Foot arch reconstruction

## Abstract

**Background:**

Müller–Weiss disease (MWD), a rare dysplastic disorder of the foot, is characterized by deformity, sclerosis, and fragmentation of the lateral part of navicular bone. Arthrodesis is the mainstay treatment for MWD. Generally, arthrodesis can be achieved through internal fixation with metallic implants, and morselized chip bone may be packed into the gap for better bone union. However, with this procedure, the original foot size is not maintained and support for the foot arch is not provided. Sequela of short foot, or flatfoot is commonly observed even though these complications of surgery had not been reported with cases of MWD treated by arthrodesis. Herein, we present a retrospective analysis of treating MWD through midfoot and hindfoot arthrodesis combined with strut allograft.

**Methods:**

From August 2006 to June 2019, 20 patients with MWD (mean age, 59.6 years; range, 40–80 years) underwent midfoot and hindfoot arthrodesis with strut bone allograft and were followed for at least 24 months. The patients were able to ambulate and participate in rehabilitation programs 3 months postoperatively.

**Results:**

The used four radiographic parameters (Meary’s angle in anteroposterior and lateral view, talonavicular coverage angle, calcaneal pitch) demonstrated significant differences (*P* < .05) preoperatively and postoperatively, but those between the postoperative values and the values at the last follow-up session did not, indicating that strut allograft was able to maintain normal alignment. The mean American Orthopaedic Foot & Ankle Society Ankle-Hindfoot scores at 2 years postoperatively revealed significant improvement from baseline, from 60.2 to 84.2 (*P* < .05). The 12-item Short Form Health Survey scores also improved significantly (*P* < .05). All patients reported substantial pain relief and exhibited improved functional outcomes and gait patterns.

**Conclusions:**

For advanced-stage MWD, arthrodesis with a precisely shaped, size-matched strut allograft provided strong support for biomechanical alignment and enhanced functional performance.

## Background

Müller–Weiss disease (MWD) is a rare foot pathology that often affects both feet. Incidence is higher among women in the fourth through sixth decades [1, 2]. The etiology of MWD is unclear, and different theories have been posited, including osteonecrosis, osteochondritis, repeated navicular stress fracture, and delayed ossification of navicular bone [1, 3]. The suspected predisposing factors or other diseases needed for differential diagnosis include rheumatoid arthritis, polyarthritis, lupus erythematosus, and chronic renal insufficiency [4, 5]. MWD is characterized by deformity, sclerosis, and fragmentation of the lateral part of the tarsal navicular bone. Patients often suffer chronic and progressive midfoot and hindfoot pain. Surgical management may be indicated and considered if symptoms persist after an adequate trial of initial conservative treatment [6].

Surgical intervention for MWD has evolved, and various procedures have been developed [3, 5, 7]. Arthrodesis, including talonavicular arthrodesis (TNA), talonavicular-cuneiform arthrodesis (TNCA), double arthrodesis, or triple arthrodesis, is indicated for perinavicular arthritis based on symptom severity and remains the mainstay treatment for MWD [8].

Generally, arthrodesis achieved through internal fixation may involve the use of screws or plates following full fusion bed preparation and decortication of the subchondral bone. Morselized chip bone is sometimes packed into the gap for better bone union. However, these procedures may lead to short foot or even flatfoot due to the collapse of the foot arch. Even though complications, caused by bony fragment resorption, nonunion, malalignment, or other postoperative outcomes, are rarely reported, flatfoot or even recurrent paradoxical pes planus varus is still observed in the daily practice. Tricortical iliac autograft had been used and proved to provide sufficient support for the foot arch. However, the patients may suffer from surgical wound pain and risks of donor site infection [9, 10].

In this study, we analyzed the clinical outcomes and midterm functional performance of patients with MWD treated with midfoot and hindfoot arthrodesis combined with strut allograft.

## Methods

### Patient profiles

**Fig. 1:**
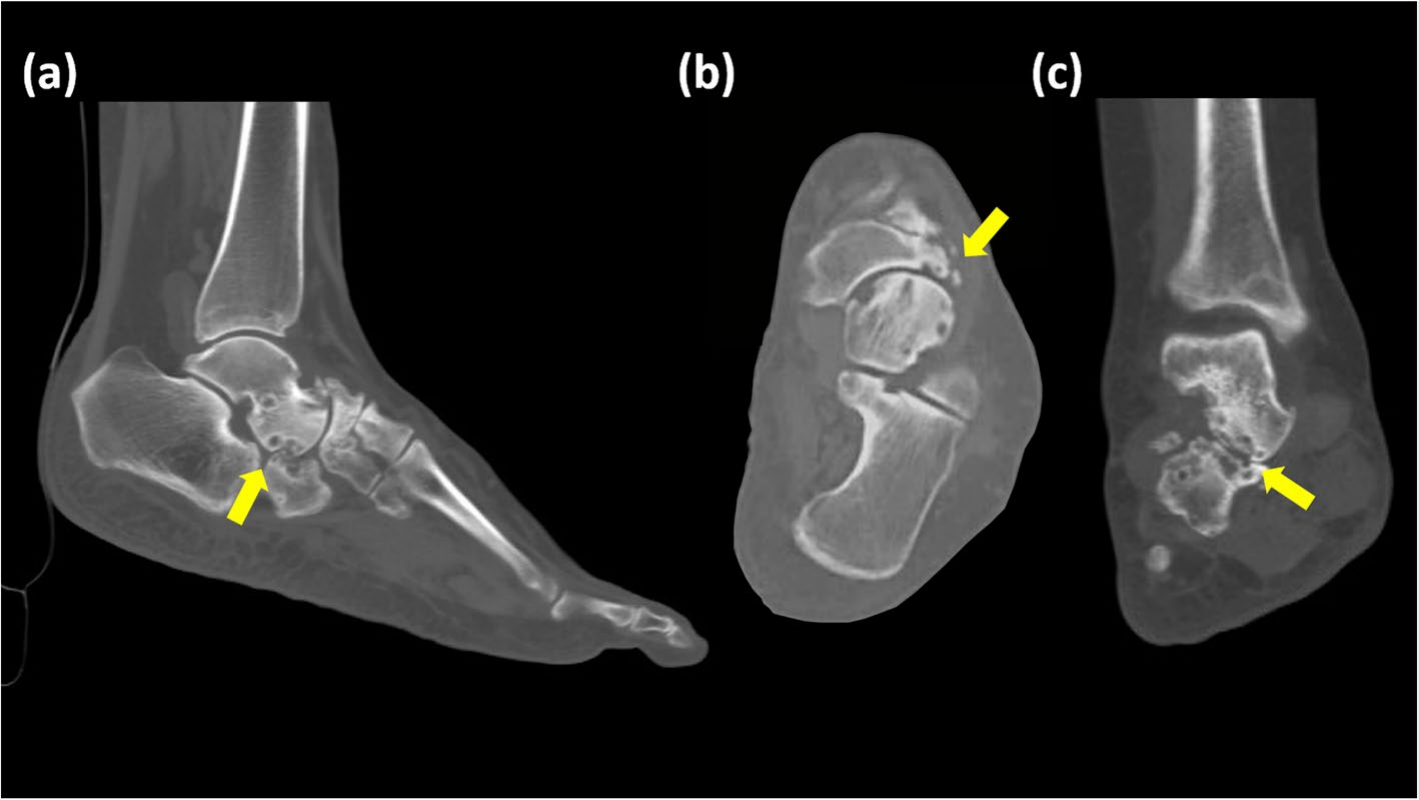
Preoperative computed tomography. **a** Sagittal view. Subtalar osteoarthritic change with subchondral cyst formation. **b** Axial view. Navicular bone osteonecrosis with fragmentation on the lateral side. **c** Coronal view. Subtalar and navicular destruction with sclerotic change

From August 2006 to June 2019, 20 consecutive patients with MWD underwent midfoot and hindfoot arthrodesis with strut allograft and were followed for a minimum of 24 months. The patients initially received conservative treatment, including nonsteroidal anti-inflammatory drugs, activity limitation, and foot orthosis protection. Surgical interventions were considered if the symptoms persisted without improvement for more than 6 months. The exclusion criteria were local infection, cellulitis, or poor lower limb circulation. Physical examination revealed considerable midfoot and hindfoot pain and limited range of motion (ROM), accompanied by local swelling and tenderness over the talonavicular joint area. Radiographs indicated the typical comma-shaped navicular bone, and computed tomography (CT) was arranged for advanced imaging studies of perinavicular osteoarthritic change, subchondral bone cyst formation, or sclerotic change (Fig. 1). The size of the necrotic part of the tarsal navicular bone is determined by CT preoperatively, and the clinical observation of the necrotic pathology during the surgery.

### Surgical technique

**Fig. 2:**
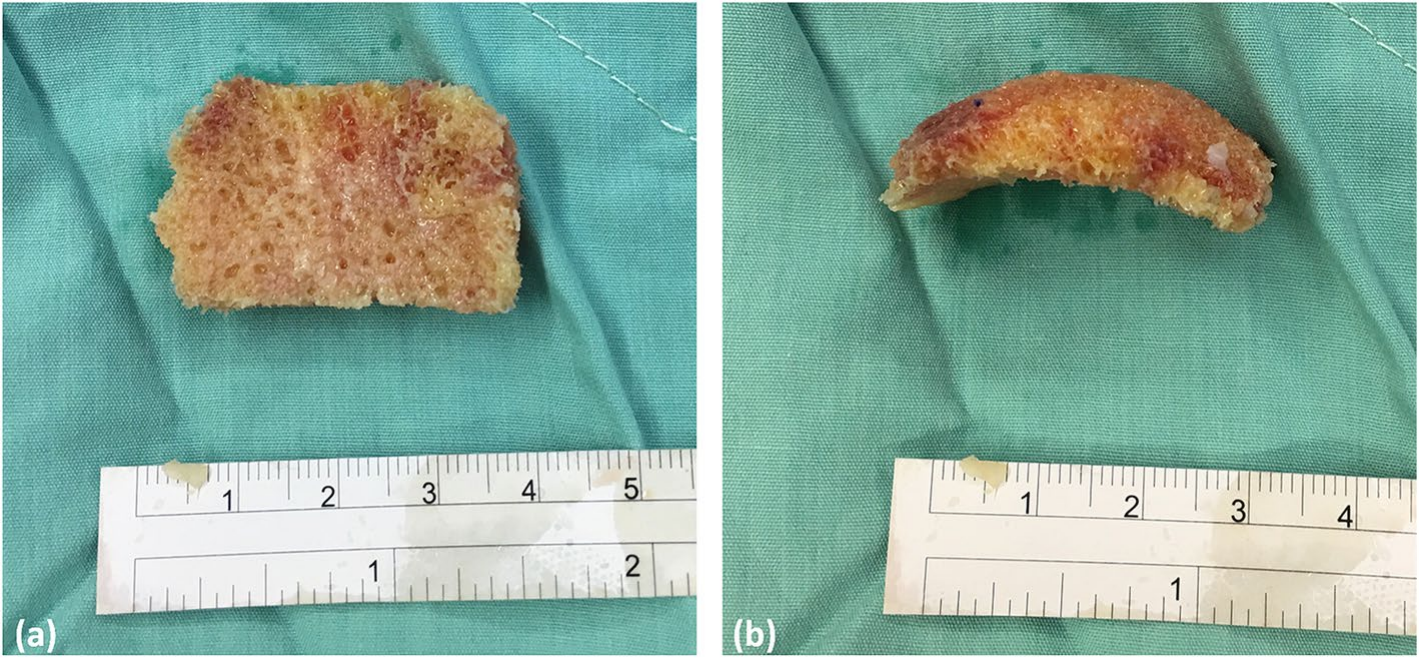
Strut bone allograft. **a** and **b** Femoral head allograft can be shaped into a size-matched bone graft mimicking the curved contour of the navicular bone

Under spinal anesthesia or endotracheal tube general anesthesia, patients were placed on the operating table in supine position. A pneumatic tourniquet was applied in all cases. The surgical site was disinfected and draped as usual. After exsanguination, one skin incision was made over the talonavicular joint on the medial aspect of the foot. The articular surface of the talonavicular joint was exposed, and the subchondral bone was excised. Another incision wound at the lateral dorsum of the foot may be needed if further exposure of the navicular bone is necessary. The necrotic part of the navicular bone was removed using a microsaw (Stryker Corp., Kalamazoo, MI, USA) and a 4-mm fluted ball power burr (Anspach Effort Inc., Palm Beach Gardens, FL, USA). With the foot kept in neutral position, the bony defect of the navicular bone was measured to ensure that the strut allograft was of best-fit size (Fig. 2). A size-matched strut allograft with fitting configuration was excised from the femoral head allograft or navicular bone allograft. Next, the bony defect of the navicular bone was filled with the strut allograft. The same strut bone grafting procedure was conducted if arthrodesis was simultaneously required for advanced subtalar or calcaneocuboid arthritis, through the traditional lateral approach from the fibular tip toward the fourth toe. For midfoot and hindfoot fixation, including TNA, TNCA, triple arthrodesis, or double arthrodesis combined with interpositional arthroplasty of the calcaneocuboid joint, screws or plates were used for securing. TN and calcaneocuboid arthrodesis could be reached by using locking plate (UPS 3.5 system) (Wright Medical Technology Inc., Arlington, TN, USA) and/or 3.0 cannulated screws (Multi-Use Compression screws) (Wright Medical Technology Inc., Arlington, TN, USA) for fixation, and subtalar fusion by using 7.0 cannulated screws (Multi-Use Compression screws) (Wright Medical Technology Inc., Arlington, TN, USA). The stability and alignment of the construct was checked under C-arm. Chips of cancellous bone from the excised bony fragments and the residual strut allograft were inserted into bone gaps. After thorough irrigation and hemostasis, two 6Fr mini-vac drains (Pacific Co., Ltd., Miaoli, Taiwan) were inserted. The wounds were closed in layers. A short leg splint was applied for external protection for 4 weeks. All surgical procedures were performed by a single orthopedic surgeon. Walking boot wearing was started 1 month postoperatively with toe-touch weight bearing, and individualized rehabilitation was provided. Patients were encouraged to walk freely with high-ankle shoe protection beginning 3 months postoperatively and to resume previous daily activities at 4 months postoperatively.

### Surgical outcomes and radiographic evaluations

**Fig. 3:**
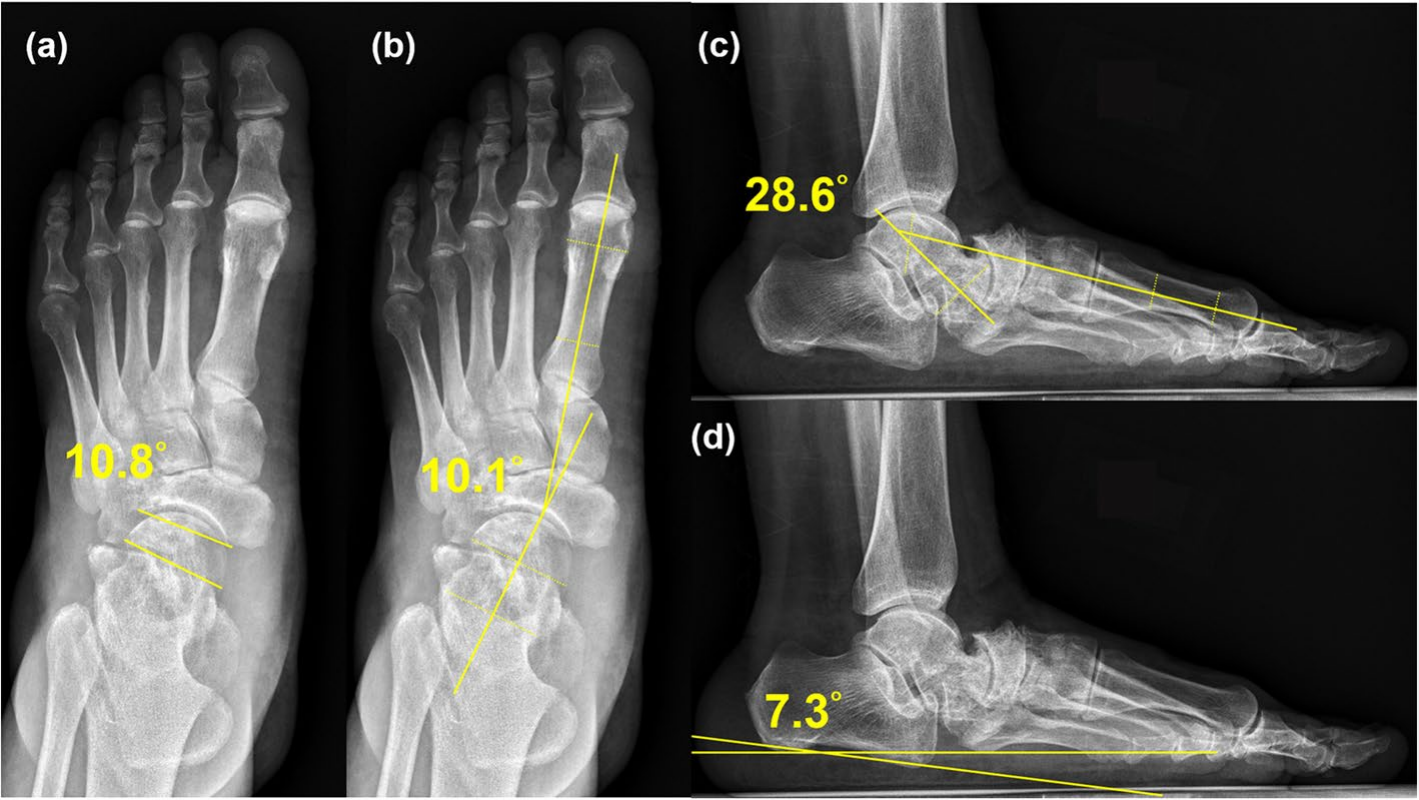
Preoperative radiography (weight-bearing view). **a** TNC angle: 10.8°. **b** and **c** Meary’s angle in AP view: 10.1°, in lateral view: − 28.6°. **d** Calcaneal pitch − 7.3°. TNC: talonavicular coverage, AP: anteroposterior

Radiographic parameters for midfoot and hindfoot misalignment and foot arch (calcaneal pitch, talonavicular coverage [TNC] angle, Meary’s angle in anteroposterior [AP] and lateral view; Fig. 3) were compared as measured from plain radiographs taken preoperatively, postoperatively, and at follow-up. The union time of the strut allograft was determined by the follow-up radiographs, with presence of bridging callus at 2–3 out of 4 cortices on AP and lateral views. Clinical outcomes were assessed using the visual analog scale (VAS), American Orthopaedic Foot & Ankle Society (AOFAS) Ankle-Hindfoot Score, and the 12-item Short Form Survey (SF-12) [11]. All patients categorized according to Maceira classification and all radiographic parameters were recorded and measured by a single orthopedic surgeon [1].

### Statistical analysis

The paired *t* test was performed to evaluate each radiographic parameter (calcaneal pitch, TNC angle, and Meary’s angle) and clinical functional outcomes (VAS, AOFAS Ankle-Hindfoot score, and SF-12) preoperatively, postoperatively, and at the last follow-up session. Significant differences were defined as *P* < .05. All data were analyzed using IBM SPSS Statistics for Windows, version 25 (IBM Corp., Armonk, NY, USA).

## Results

### Patient demographics

A total of 20 patients (4 male, 16 female) with a mean age of 59.6 ± 15.6 years (range: 30–80 years) were enrolled (Table 1). Zero (0%), four (20%), six (30%), four (20%), and six (30%) patients were categorized into stages I, II, III, IV, and V of the Maceira classification, respectively [1]. The average body mass index was 25.9 ± 3.53 kg/m^2^. In all, 20 strut allografts were used (18 femoral heads and 2 navicular bones). Seven patients received TNA and subtalar fusion, four patients received double arthrodesis with interpositional arthroplasty of the calcaneocuboid joint, and nine patients received triple arthrodesis. The estimated blood loss during surgery was minimal. The average union time of bone allograft uptake was 4.0 ± 1.6 months, and the mean follow-up duration was 31.4 ± 7.0 months.

**Table 1:** Patient Demographics and Perioperative Variables

Patients	20 (4 M, 16 F) ^**†**^				
Maceira classification (%)	I	II	III	IV	V
	0	4	6	4	6
Age (years)	59.6 ± 15.6				
BMI (kg/m^2^)	25.9 ± 3.53				
Operative site ^**‡**^	10 left/10 right				
Allograft	18 femoral heads/2 navicular bones				
NWB duration (months)	3 (postoperative protocol)				
Follow-up duration (months) *	31.4 ± 7.00				
Union period (months)	4.02 ± 1.61				

Data are presented as means ± standard deviations.

^**†**^ *M* male, *F* female

* *NWB* non–weight bearing

### Radiographic evaluations

Table 2 presents the radiographic comparisons of the patients preoperatively and postoperatively. The calcaneal pitch was 4.23° ± 10.5° preoperatively and 17.0° ± 4.62° postoperatively (*P* < .001). The TNC angle was 18.5° ± 10.1° preoperatively and 5.12° ± 1.56° postoperatively (*P* < .001). Thus, the differences for both calcaneal pitch and TNC angle were significant. The Meary’s angles differed significantly preoperatively (14.2° ± 6.72° in anteroposterior [AP] view and − 4.45° ± 14.1° in lateral view) and postoperatively (3.43° ± 1.88° in AP view and 2.71° ± 1.63° in lateral view; *P* < .001 and *P* = .04, respectively). This indicates that the foot deformity was reduced and corrected anatomically.

**Table 2:** Comparison of Preoperative, Postoperative, and Follow-Up Radiographic Parameters

	Preoperative	95% CI	Postoperative	95% CI	Follow-up
Meary’s angle AP view ^**†**^	14.2° ± 6.72°	11.3° – 17.2°	3.43° ± 1.88° *	2.60° – 4.25°	3.66° ± 1.85°
Meary’s angle Lateral view	−4.45° ± 14.1°	−10.6° – 1.74°	2.71° ± 1.63° *	2.00° – 3.43°	2.65° ± 1.23°
TNC angle ^**‡**^	18.5° ± 10.1°	14.1° – 23.0°	5.12° ± 1.56° *	4.43° – 5.80°	5.42° ± 1.58°
Calcaneal pitch	4.23° ± 10.5°	−0.36° – 8.82°	17.0° ± 4.62° *	15.0° – 19.0°	18.3° ± 4.86°

Data are presented as means ± standard deviations.

^**†**^ *AP* anteroposterior

^**‡**^ *TNC* talonavicular coverage

* indicates significance at *P* < .05

*CI* Confidence interval.

Furthermore, no significant differences were observed in comparisons of the postoperative and follow-up radiographic parameters, whether for calcaneal pitch (17.0° ± 4.62° vs. 18.3° ± 4.86°, *P* = .322), TNC angle (5.12° ± 1.56° vs. 5.42° ± 1.58°, *P* = .147), or Meary’s angle (AP view 3.43° ± 1.88° vs. 3.66° ± 1.85°, *P* = .089; lateral view 2.71° ± 1.63° vs. 2.65° ± 1.23°, *P* = .760). These results substantiate that the strut allograft can maintain the corrected foot architecture in the midterm followup.

**Fig. 4:**
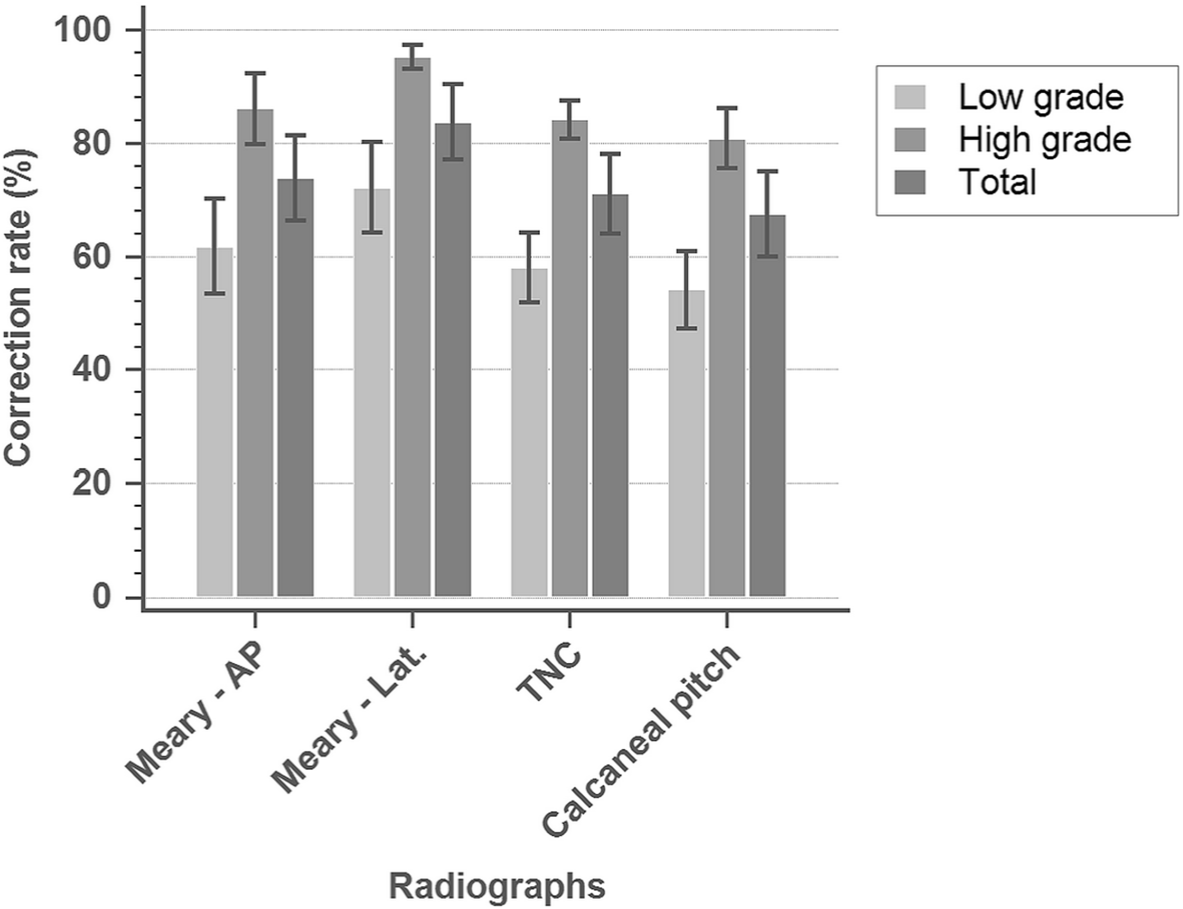
Correction rates for each radiographic parameter were at least 50% in the low-grade group and at least 80% in the high-grade group. * indicates significant differences between correction rates in the low- and high-grade groups.

Under the Maceira classification, the patients were divided into 2 groups: a low-grade group (stage II or III) and a high-grade group (stage IV or V; Fig. 4). The correction rates of all radiographic parameters in the low-grade group indicated at least 50% improvement, with an AP Meary’s angle of 61.7% ± 11.7%, a lateral Meary’s angle of 72.1% ± 11.1%, a TNC angle of 58.0% ± 8.72%, and a calcaneal pitch of 54.1% ± 9.48%. In the high-grade group, all radiographic parameters indicated up to 80% improvement in correction (AP Meary’s angle 86.0% ± 8.84%, lateral Meary’s angle 95.2% ± 3.00%, TNC angle 84.1% ± 4.74%, calcaneal pitch 80.0% ± 7.37%), with significant differences compared with the low-grade group.

### Clinical outcome assessment

For clinical outcome assessment, we used the VAS, AOFAS Ankle-Hindfoot score, and SF-12 as indexes (Table 3). The mean VAS score was 5.30 ± 1.42 preoperatively and 1.30 ± 1.17 postoperatively (*P* < .001). The total AOFAS Ankle-Hindfoot score was 60.2 ± 9.21 preoperatively and 84.2 ± 12.2 postoperatively (*P* < .001). Moreover, significant differences were noted in the subclassifications of the AOFAS Ankle-Hindfoot scores, specifically in the pain score (19.5 ± 9.45 vs. 33.0 ± 6.57, *P* < .001), function score (33.5 ± 6.22 vs. 38.7 ± 6.88, *P* = .011), and alignment score (6.15 ± 5.29 vs. 9.25 ± 1.68, *P* = .014). Regarding the SF-12 scoring system, the mean physical score differed significantly preoperatively and postoperatively (39.4 ± 6.88 vs. 51.4 ± 6.08; *P* < .001), as did the mental score (53.4 ± 9.65 vs. 57.5 ± 7.36; *P* = .03) and total SF-12 score (95.1 ± 12.0 vs. 107 ± 12.6; *P* < .001). Significant preoperative–postoperative differences were observed in the mean VAS, AOFAS Ankle-Hindfoot score (and all subcategories), and SF-12 scores, indicating substantial improvements in pain relief, functional activity, and physical and mental health after the operation.

**Table 3:** Comparison of AOFAS Ankle-Hindfoot Scores

	Preoperative evaluation	95% CI	Postoperative evaluation	95% CI	*P* value
Total AOFAS score	60.2 ± 9.21	56.2–64.2	84.2 ± 12.2	78.8–89.5	< .001
AOFAS pain	19.5 ± 9.45	15.4–23.6	33.0 ± 6.57	30.1–35.9	< .001
AOFAS function	33.5 ± 6.22	30.8–36.3	38.7 ± 6.88	35.7–41.7	.011
AOFAS alignment	6.15 ± 5.29	3.83–8.47	9.25 ± 1.68	8.51–9.99	.014
Total SF-12	95.1 ± 12.0	89.9–100	107 ± 12.6	101–112	< .001
Physical	39.4 ± 6.88	36.4–42.4	51.4 ± 6.08	48.8–54.1	< .001
Mental	53.4 ± 9.65	49.2–57.7	57.5 ± 7.36	54.3–60.7	.03
VAS score	5.30 ± 1.42	4.68–5.92	1.30 ± 1.17	0.79–1.81	< .001

Data are presented as means ± standard deviations.

*CI* confidence interval.

### Complications

Among the patients who underwent hindfoot arthrodesis with strut allograft (Table 4), none experienced delayed or poor wound healing, or infection. One patient was found to have implant failure with screw breakage without any discomfort or limitation of ROM. However, 2 of the 20 patients (10%) reported foot discomfort at the most recent follow-up session. After detailed inquiry for differential diagnosis, osteoarthritic change was identified as the cause of fourth tarsometatarsal joint pain in one patient, and another patient reported experiencing discomfort over the fifth metatarsophalangeal joint. Foot discomfort in both patients were therefore not related to the surgery.

**Table 4:** Postoperative Complications

Delayed/poor wound healing	0
Infection	0
Pain/swelling/stiffness	2
Implant failure	0
Screw loosening	0
Implant breakage	0
Recurrence/deformity	0
Gait disturbance	0
Total	2

Number: the number of each complication.

The patients returned to our clinic 2 weeks postoperatively and then regular follow-up. No previous talonavicular joint pain was mentioned at postoperative 2 months, but a mild sensation of discomfort over the surgical wound was noted. All patients needed no longer to take prescription or over the counter medications. Also, the patient-reported outcomes indicated satisfactory results with favorable activity function. These results convincingly demonstrate the favorable application of strut allograft, especially for advanced MWD.

However, two patients were found to experience foot discomfort at the most recent follow-up session. A possible reason for the later appearance of adjacent joint osteoarthritis may be that the fusion procedure hindered the eversion and inversion of the subtalar joint, increasing biomechanical stress over the lateral tarsometatarsal and metatarsophalangeal joint (as with adjacent segment disease after spinal fusion surgery). The discomfort of these two patients was reduced after shoe modification with insoles, and no additional surgical intervention was required. Furthermore, all patients presented notable improvements in gait pattern and shoe fit, without flatfoot or recurrent deformity. The detailed demographic data was listed in Table 5.

**Table 5:** Patient with Müller-Weiss disease, preoperative staging (Maceira classification), image evaluation and postoperative fixation device as well as bone union time

Patient	Gender	Age	Side	Maceira stage	Pre-operative radiographs (degrees)			Bone graft
					Meary angle (AP/lateral)	TNC angle	Calcaneal pitch	
1	F	48	Left	II	5.28 / 13.55	4.53	14.18	Allograft
2	F	72	Left	V	30.93 / -23.07	38.18	−8.3	Allograft
3	M	40	Right	V	16.76 / -21.02	24.29	−16.41	Allograft
4	F	56	Left	III	11.97 / 5.23	10.77	13.85	Allograft
5	M	30	Right	II	8.22 / 14.84	6.79	14.84	Allograft
6	F	65	Right	II	9.82 / 16.88	7.32	15.03	Allograft
7	F	72	Right	II	3.19 / 12.58	4.81	16.87	Allograft
8	M	72	Right	IV	15.97 / -5.65	23.21	0.39	Allograft
9	F	68	Left	III	11.41 / -2.54	10.67	11.7	Allograft
10	F	80	Right	V	29 / -25.71	38	−9.06	Allograft
11	F	51	Left	III	10.91 / 4.07	15.2	9.46	Allograft
12	F	70	Left	V	17.16 / -16.62	23.64	−6.37	Allograft
13	F	61	Left	IV	14.09 / -7.86	17.13	2.54	Allograft
14	F	46	Left	V	17.24 / -26.74	25.32	−8.05	Allograft
15	F	68	Right	III	10.61 / 6.94	14.51	11.24	Allograft
16	F	68	Right	III	14.02 / -3.91	17.54	7.12	Allograft
17	F	64	Left	IV	15.62 / -4.47	18.41	8.12	Allograft
18	F	65	Right	V	19.22 / -17.75	33.98	−11.59	Allograft
19	F	67	Left	III	11.73 / 4.49	16.57	10.28	Allograft
20	M	19	Right	VI	11.8 / -12.44	19.84	8.7	Allograft
Patient	Surgery		Implants		Follow-up (months)	Bone union (months)		Complications
1	TNA + subtalar fusion		Locking plate + cannulated screws		30.9	2.9		–
2	Triple arthrodesis		Locking plates + cannulated screws		28.3	4.3		–
3	Triple arthrodesis		Locking plates + cannulated screws		25.5	7.0		Pain over fourth TMTJ
4	TNA + subtalar fusion		Locking plate + cannulated screws		53.1	2.9		–
5	TNA + subtalar fusion		Locking plate + cannulated screws		32.6	2.8		–
6	TNA + subtalar fusion		Locking plate + cannulated screws		41.4	3.1		–
7	TNA + subtalar fusion		Locking plate + cannulated screws		27.2	2.8		–
8	Double arthrodesis + interpositional arthroplasty		Locking plate + cannulated screws		35.1	3.2		–
9	Double arthrodesis + interpositional arthroplasty		Locking plate + cannulated screws		34.1	3.5		–
10	Triple arthrodesis		Locking plates + cannulated screws		25.8	1.8		Pain over fifth MTPJ
11	TNA + subtalar fusion		Locking plate + cannulated screws		38.3	3.3		–
12	Triple arthrodesis		Locking plates + cannulated screws		28.3	2.9		–
13	Triple arthrodesis		Locking plate + cannulated screws		29.1	5.1		–
14	Triple arthrodesis		Locking plate + cannulated screws		34.9	4.9		–
15	TNA + subtalar fusion		Locking plate + cannulated screws		30.7	6.7		–
16	Double arthrodesis + interpositional arthroplasty		Locking plate + cannulated screws		26.9	7.9		–
17	Triple arthrodesis		Locking plates + cannulated screws		24.1	3.0		–
18	Triple arthrodesis		Locking plates + cannulated screws		24.9	4.8		–
19	Double arthrodesis + interpositional arthroplasty		Locking plate + cannulated screws		24.1	4.4		–
20	Triple arthrodesis		Locking plates + cannulated screws		32.9	2.9		–

*AP* anterior-posterior.

*TNC* Talo-calcaneal coverage.

## Discussion

Arthrodesis is regarded as the mainstay treatment for MWD. Several surgical techniques have been proposed for different types of MWD according to the Maceira classification, including simple excision with drilling for decompression [8], TNA, TNCA [3], double fusion and triple arthrodesis [12]. Favorable clinical outcomes have been reported for these treatments in case reports and retrospective studies [3, 7, 12]. Based on current knowledge, direct fusion without a bone graft may result in foot deformity such as flatfoot or even recurrent paradoxical pes planus varus foot, especially in advanced MWD, leading to poor shoe fit and disturbances in gait pattern. Morselized bone graft has been widely used to promote bone healing in various orthopedic surgery [13, 14]. The drawbacks are that the chip bone cannot provide adequate structural support and that the mechanical stability is solely dependent on the application of metallic implants [15, 16]. In case of the resorption of bone graft without sufficient bone union, the fixation configuration may be destroyed and the implants may be broken, thus leading to the loss of foot arches and alignment and subsequent foot deformity [17]. A strut bone grafting procedure has been demonstrated to improve bone healing and maintain foot alignment, but results remain inconsistent in the literature [18–20]. Consensus has not yet been reached with regard to the optimal surgical procedure for treating MWD; arthrodesis with or without bone graft and type of bone graft (autograft vs. allograft, structural vs. morselized) remains a case-by-case determination. In this case series, we analyzed the clinical outcomes and functional performance of patients diagnosed as having MWD and treated by arthrodesis with strut allograft.

Using strut bone graft has gradually become a trend in various types of foot and ankle surgery. In 1996, Amendola et al. presented a case series of 15 consecutive patients experiencing hindfoot pain due to calcaneal fracture. They were treated with subtalar arthrodesis combined with interpositional grafts of the iliac crest [21]. The results indicated significant improvements; specifically, significant preoperative–postoperative differences were observed in the AOFAS Ankle-Hindfoot scale and VAS score (*P* < .001). Radiographic examination revealed a 100% arthrodesis union rate with the restoration of heel height. The researchers described how the use of structural bone graft allowed optimal arthrodesis and correction of calcaneal collapse simultaneously. Regarding foot and ankle reconstruction, Ayerza et al. reported favorable structural allograft survival rates of 79% at 5 and 10 years after tumor resections for 44 pathologies [22]. The large bone defect was sufficiently reconstituted to the previous anatomical configuration. Only three complications were reported, two involving graft fracture and one involving nonunion.

Similar concepts can be applied in MWD treatment. Gaps created by the removal of the necrotic part of the navicular bone are often filled with morselized chip bone, which enables the promotion of bone growth and fusion [23]. However, the morselized chip bone often fails to provide adequate mechanical support for foot arch reconstruction. In 2019, Ahmed et al. reviewed 10 studies to analyze the efficacy and effectiveness of surgical management in treating MWD [2]. In four of the studies, patients were treated with arthrodesis combined with tricortical iliac autograft. The results were clinically satisfactory, with significant differences between preoperative and postoperative AOFAS Ankle-Hindfoot scores. Structural bone grafts were able to maintain the reconstructed foot arches. A similar concept is supported by the results of another study. Levinson et al. proposed a novel operative technique involving the debridement of necrotic navicular bone followed by a free vascularized bone graft of the medial femoral condyle [24]. At 18-month follow-up, the patient presented excellent outcomes and a return to previous levels of function, as indicated by SF-12 scores of 73.5 preoperatively and 117.4 postoperatively.

**Fig. 5:**
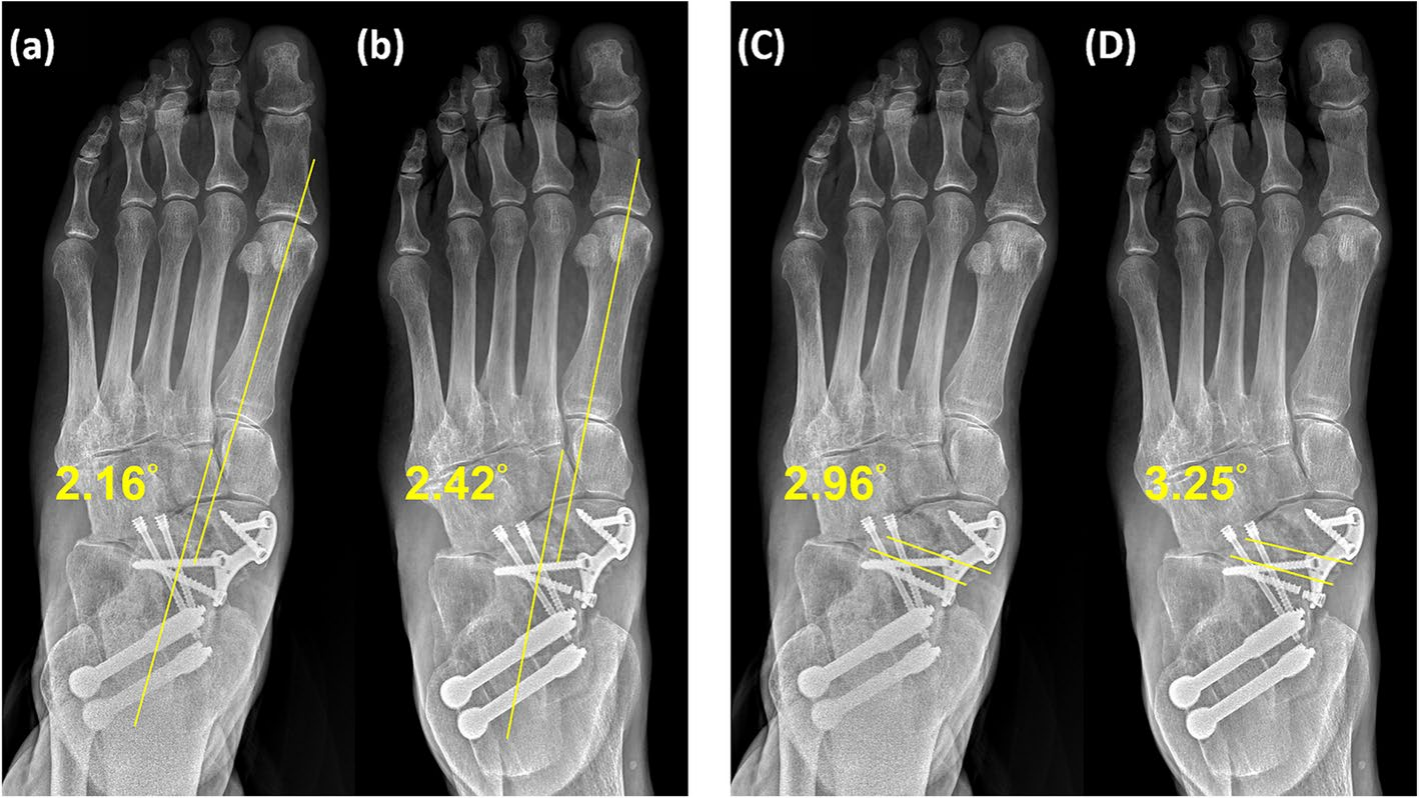
Foot AP radiography (weight-bearing view). **a** Postoperative Meary’s angle: 2.16°. **b** Follow-up Meary’s angle: 2.42°. **c** Postoperative TNC angle: 2.96°. **d** Follow-up TNC angle: 3.25°

**Fig. 6:**
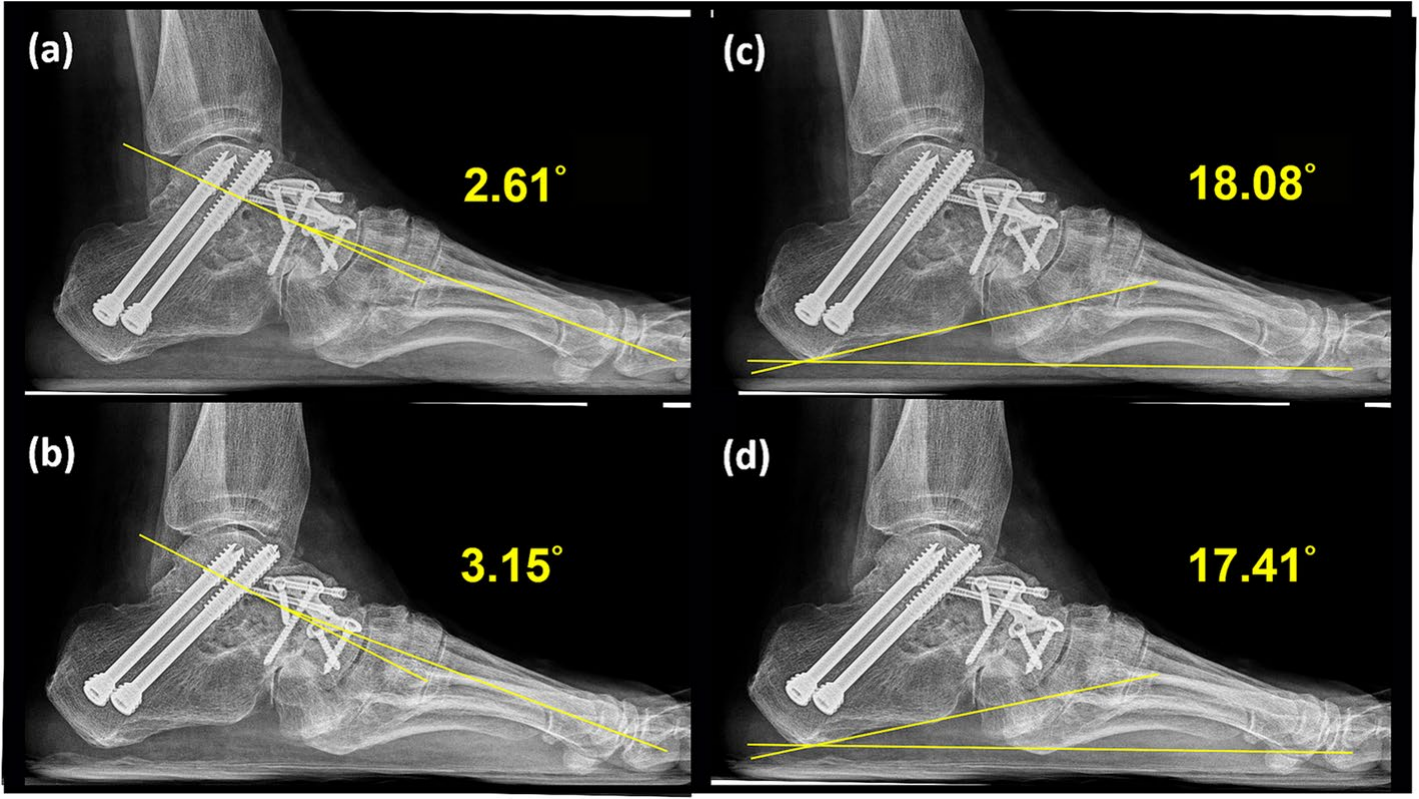
Ankle lateral radiography (weight-bearing view). **a** Postoperative Meary’s angle: − 2.61°. **b** Follow-up Meary’s angle: − 3.15°. **c** Postoperative calcaneal pitch: 18.08°. **d** Follow-up calcaneal pitch: 17.41°

In the present study, the preoperative and postoperative radiographic parameters differed significantly, indicating that strut allograft anatomically reduced the foot and reconstructed the alignment. Notably, no significant difference was observed between the radiographic parameters after the operation and at 2-year follow-up (Figs. 5 and 6). No allograft resorption subsidence, or sclerosis was noted. These results support the premise that structural bone graft can provide the foot arch with robust support.

In recent decades, the use of bone allograft has become increasingly common, including for fracture, musculoskeletal tumor, joint instability or arthritic change, deformity correction, and reconstruction. The efficacy and effectiveness of bone allograft use have been confirmed [25, 26]. Coetzee et al. reported a case series of 44 patients with large talar body defects (after a failed ankle arthroplasty or avascular necrosis) undergoing ankle arthrodesis or tibio-talo-calcaneal fusion by using femoral head allografts [27]. The overall satisfaction rate was 78.6%, with 90.7% of ankles exhibiting favorable fusion at an average of 18.7 weeks. The researchers concluded that the use of femoral head allograft to treat large talar defects is viable not only for maintaining limb length but also for preventing collapse in the corrected foot. Chu et al. conducted a case series of 25 consecutive patients with osteochondral lesions of the talus treated by fresh-frozen allograft transplantation [28]. The midterm results indicated significant improvement in both AOFAS Ankle-Hindfoot scores and SF-12 scores. No bone allograft collapse or revision was observed in the final follow-up session, indicating that fresh-frozen allograft transplantation is an option in treating symptomatic osteochondral lesions of the talus.

Tricortical iliac autografts constitute most structural bone grafts used in treating MWD [29]. Although such autografts help maintain the alignment of feet, potential complications remain, including a limited amount of bone availability, increased risk of donor site infection, and surgical wound pain at the donor site [9, 10]. The use of bone allografts may circumvent these drawbacks. Tan et al. published a case report in which MWD was treated by TNC fusion with a femoral head allograft [30]. Specifically, a femoral head allograft was carefully remodeled to fit the defect after the removal of the cartilage and necrotic bone. The patient was fully weight bearing 6 months postoperatively and at 10 months postoperatively was able to walk pain free. Follow-up radiographs taken 14 months postoperatively indicated favorable consolidation without any signs of osteolysis. Another case report proposed by Nelson et al. demonstrated a successful medial arch fusion by using tricortical iliac crest allograft at 38 months of follow-up without any postoperative symptoms [31]. Therefore, the researchers concluded that allografts may constitute a safe and effective operative option for the treatment of MWD. A femoral head allograft can be remodeled to match the shape of the defect precisely; it is also associated with no donor site morbidity. We arrived at a similar conclusion in the present study. All bone grafts were strut allografts; specifically, 2 were navicular allografts and 18 were femoral head allografts. The implanted bone allograft achieved favorable bone healing, and patients reported satisfactory functional outcomes. Using bone allograft can remove the need for graft harvest from asymptomatic sites and prevent donor site morbidity.

General concerns in using bone allograft transplantation include disease transmission, immune response leading to graft rejection, and the development of malignancy from the graft, and the potential of increased risks of infection, especially in higher risk patients such as diabetics [32, 33]. Regarding disease transmission, although the incidence of bacterial infection is low, graft-related infections have been reported [34–36]. According to the American Association of Tissue Banks, with an adequate processing protocol, the potential for disease transmission or graft contamination can be reduced such that it is an acceptable risk in general orthopedic surgery [37]. In consideration of immune response, the bone graft preservation method should be carefully selected. According to the methods of preparation and storage used in orthopedic surgery, bone allografts can be subclassified as freeze dried, fresh frozen, and fresh cold stored [32]. Fresh cold stored bone allograft, known for its favorable osteoinductive potential with regard to the indwelling bone morphogenetic protein (BMP) and high cellular viability, is most commonly used in cartilage grafting. However, the high risk of disease transmission and immunogenicity constitute serious concerns [38]. Freeze-dried bone allografts, with pure osteoconductive potential by complete BMP depletion, have the lowest immunogenicity and the lowest likelihood of viral transmission. Fresh-frozen bone allografts, preserving partial BMP potential, have demonstrated lower immunogenicity than fresh cold-stored bone allografts. In our series, we selected fresh-frozen bone allografts for bony defect restoration in pursuit of the benefits of proper osteoinduction and smaller immune reactions. Our results revealed favorable bone healing with adequate osteointegration and, thus far, no detrimental effects such as graft rejection or bone resorption.

**Fig. 7:**
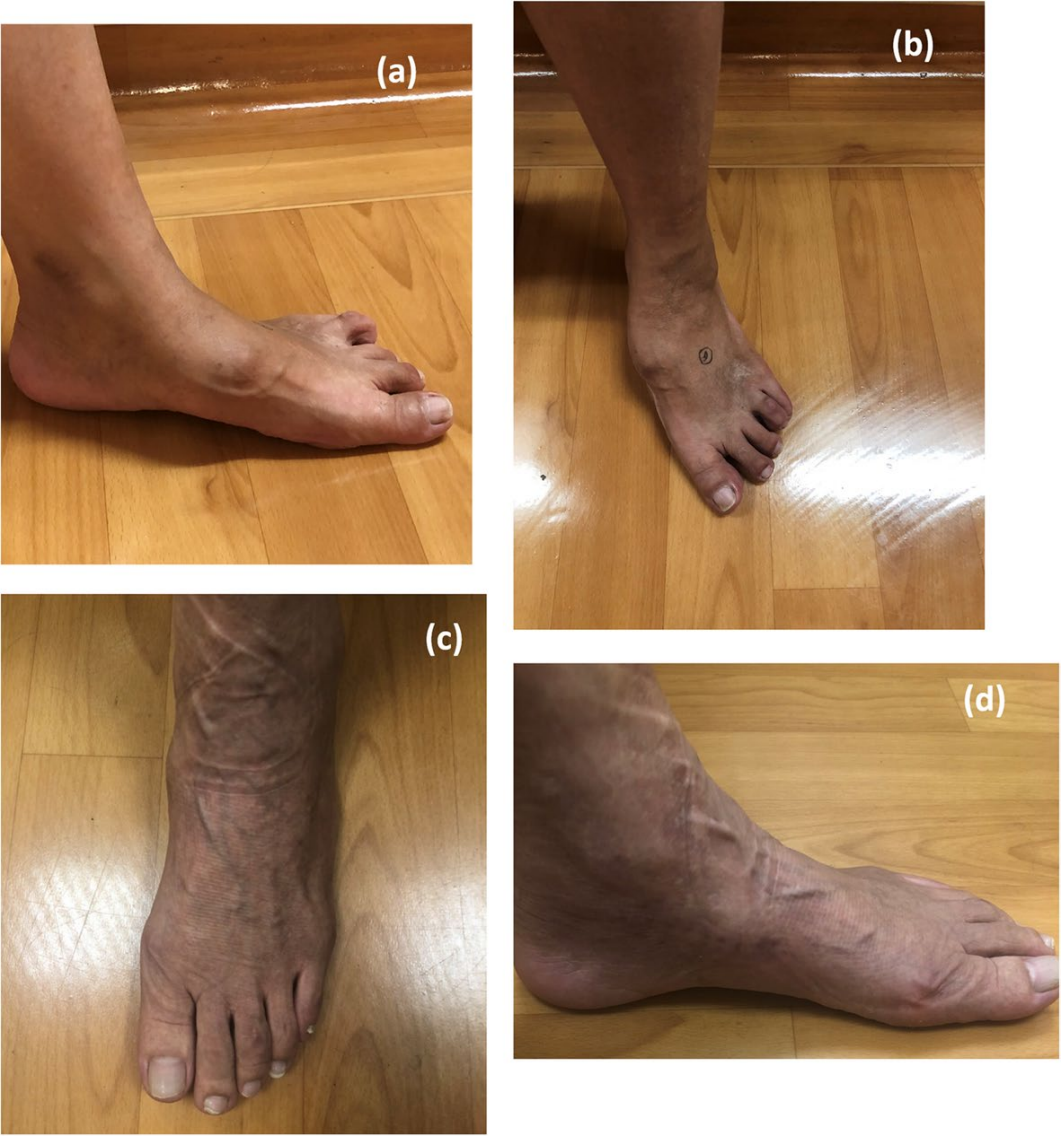
Clinical image captured preoperatively (**a**), (**b**) and 8 months postoperatively (**c**), (**d**) displaying a protruded medial midfoot bony prominence and a reconstructed foot arch without medial protrusion of the navicular bone respectively

Our study illustrates several advantages of treating MWD with bone allograft. First, defect size is not a concern according to our experience in treating patients with Müller–Weiss disease in different severity. Compared with autogenous, even when the necrotic portion involves the whole navicular bone, lesions of any shape and size can be matched by the precisely shaped bone allograft [30]. Moreover, bone allografts eliminate the requirement for graft harvest from asymptomatic sites and reduce the possibility of donor site morbidity. In addition, size-matched strut allografts were used instead of local chip bone autografts to fill the bony defect. They provided sound mechanical stability and restored the foot’s anatomic geometry (Fig. 7).

To the best of our knowledge, this study is the largest series to use strut allografts to reconstruct the foot arch in treating MWD regardless the size of the bony defect. This study has several limitations, including its retrospective nature, single-centered analysis, and small sample size. Also, all radiographic parameters and questionnaires were conducted by a single orthopedic physician. Furthermore, the study was not homogenous because the surgical method of arthrodesis, which may include talonavicular joint fusion, TNC joint fusion, triple arthrodesis, or double arthrodesis with interpositional arthroplasty, is known to be a critical determinant of functional outcomes and ROM of the hindfoot joint. Different hardware constructs were used for different types of arthrodesis. However, in view of the restoration of foot bone alignment and the mechanical axis, especially in the case of advanced MWD, the use of strut allografts for supporting the size-matched bony defect could be applicable to all these fusion procedures. The final major limitation is that no fixation in situ or autogenous bone grafting group was enrolled as a control group. MWD is a relatively rare pathology. A multicenter, retrospective comparative study may constitute a legitimate model allowing the recruitment of a large number of patients. A prospective cohort study or randomized controlled trial should be conducted as an evidence-based clinical evaluation of the efficacy and effectiveness of using strut allografts in treating MWD.

## Conclusions

This paper presents a case series in which patients were surgically treated for MWD through arthrodesis combined with strut allograft. Midterm allograft survival was favorable, graft collapse did not occur, and functional outcomes were satisfactory. For advanced MWD, arthrodesis with an individualized and precisely shaped size-matched strut allograft can provide strong support for the restoration of biomechanical alignment. A long-term evaluation with a larger sample size is warranted to address the limitations of the present study.

## Data Availability

All data generated or analyzed during this study are included in this published article.
